# Complete Genome Sequence of Streptococcus oralis 34

**DOI:** 10.1128/MRA.00760-21

**Published:** 2021-09-02

**Authors:** Justin R. Kaspar

**Affiliations:** a Division of Biosciences, The Ohio State University College of Dentistry, Columbus, Ohio, USA; Montana State University

## Abstract

Streptococcus oralis is an early colonizer and one of the most abundant species found in the human oral cavity. We report the complete genome sequence of S. oralis 34 (1,920,884 bp; GC content, 41.3%), commonly used in many oral microbiology studies exploring bacterial attachment and interaction(s) within mixed-species model systems.

## ANNOUNCEMENT

Streptococcus oralis is a Gram-positive, nonmotile, alpha-hemolytic bacterium and one of the most abundant commensal bacteria in the human oral cavity, considered to be an early colonizer of dental plaque (1, 2). S. oralis belongs to the Mitis group of streptococci (3), which contains the major human pathogens Streptococcus pneumoniae and Streptococcus mitis. Although S. oralis is associated with oral health, S. oralis can gain access to the bloodstream and cause subacute infective endocarditis (IE) as an opportunistic pathogen (4–6). In 2016, S. oralis was split into three different subspecies, S. oralis subsp. *oralis*, S. oralis subsp. *tigurinus*, and S. oralis subsp. *dentisani* (7). However, due to genetic recombination and horizontal gene transfer via natural transformation between members of the Mitis group, classification of Mitis group species is often difficult (8).

S. oralis isolate 34 was originally obtained from R. J. Gibbons at the Forsyth Dental Center in Boston, MA, and at that time was called Streptococcus sanguis 34 (9). The isolate was used frequently in studies of oral bacterial adherence, both to surfaces and to other oral bacteria (10–12). Today, S. oralis 34 is still commonly used in oral microbiology research due to its robust biofilm formation properties (13, 14) and is commonly used within mixed-species models (15–17). However, a complete genome sequence for this organism was lacking.

For whole-genome sequencing, S. oralis 34 was resuscitated from a –80°C glycerol stock by streaking onto a brain heart infusion (BHI) agar plate and grown within an incubator at 37°C, 5% CO_2_, for 48 h. Then, 5 ml of BHI broth was inoculated with the strain and grown overnight (37°C, 5% CO_2_). The following morning, genomic DNA was purified using the DNeasy PowerLyzer microbial kit (Qiagen; catalog no. 12255-50), and the concentration was determined using a Qubit Flex fluorometer (Thermo Fisher Scientific; catalog no. Q33327) and the Qubit double-stranded DNA (dsDNA) broad-range (BR) assay kit (Thermo Fisher Scientific; catalog no. Q32850). The Microbial Genome Sequencing Center (MiGS; Pittsburgh, PA) performed combined short- and long-read sequencing (Small Nanopore Combo sequencing package; Illumina and Oxford Nanopore Technologies [ONT], respectively) and *de novo* assembly. Default parameters were used except where otherwise noted. Short reads were obtained using the Illumina Nextera kit and NextSeq 550 platform (18). For ONT sequencing, libraries were prepared using the kit SQK-LSK109 to the manufacturer’s specifications (no DNA size selection/shearing), sequencing was performed on a MinION R9 flow cell, and base calling was performed using Guppy v4.2.2 (GPU mode) (19). Illumina paired-end reads (2 × 151 bp) and ONT long reads were provided as fastq files (Illumina, 1,701,038 reads, 468,658,143 bases, 244× coverage; ONT, 634,222 reads, 317,505,913 bases, 165× coverage). Quality control and adapter trimming were performed using bcl2fastq v2.20.0.445 (20) and Porechop v0.2.3_seqan.2.1.1 (21) for Illumina and ONT sequencing, respectively. Hybrid assembly with the Illumina and ONT reads was performed using Unicycler v0.4.8 (22). The assembly statistics were recorded using QUAST v5.0.2 (23). The assembly annotation was performed using Prokka v1.14.5 (24).

The S. oralis 34 sequence was deposited at GenBank as one circular contig (1,920,884 bp; GC content, 41.3%). GenBank annotated the genome sequence using the Prokaryotic Genome Annotation Pipeline (PGAP) v5.2 (25).

### Data availability.

The S. oralis 34 sequence is available in GenBank under accession no. CP079724, BioProject accession no. PRJNA746546, and BioSample accession no. SAMN20209571. The raw sequence reads are accessible under Sequence Read Archive accession no. SRX11573463 (Illumina) and SRX11573464 (MinION).
